# Clinical implications of renin–angiotensin system inhibitors for development and progression of non-alcoholic fatty liver disease

**DOI:** 10.1038/s41598-021-81959-1

**Published:** 2021-02-03

**Authors:** Kwang Min Kim, Ji-Hye Roh, Sangjin Lee, Jeong-Hyun Yoon

**Affiliations:** 1 Department of Medicine, Samsung Changwon Hospital, Sungkyunkwan University School of Medicine, Changwon, South Korea; 2 College of Pharmacy, Pusan National University, Busan, South Korea; 3 Department of Statistics, College of Natural Science, Pusan National University, Busan, South Korea

**Keywords:** Disease prevention, Gastroenterology, Non-alcoholic fatty liver disease

## Abstract

Recently, there has been an increasing interest in the therapeutic efficacy of RAS inhibitors (RASi) in patients with non-alcoholic fatty liver disease (NAFLD) because they may reduce oxidative stress, inflammatory markers, and enhanced fibrosis. An objective of this study was to investigate the role of RASi on NAFLD development and progression in a large cohort. We conducted a nested case–control study. Study subjects were classified into two study cohorts according to baseline NAFLD status: non-NAFLD (n = 184,581) and established NALFD (n = 27,565). An NAFLD development or progression case was defined as a patient with newly developed NAFLD or new progression of advanced fibrosis from non-NAFLD and established NALFD cohorts, respectively. A conditional logistic regression analysis was conducted to estimate the associations between RASi exposure and NAFLD development/progression. Overall, no significant association was evident between RASi use and NAFLD development or progression (NAFLD development; ever-user vs. never-user: OR 1.017; 95% CI 0.842–1.230, NAFLD progression; ever-user vs. never-user: aOR 0.942; 95% CI 0.803–1.105). RASi ever-use in cases of individuals who were obese or who had normal fasting plasma glucose (FPG) was associated with reduced risk of both NAFLD development (body mass index (BMI) ≥ 25 kg/m^2^: 0.708 [95% confidence interval (CI) 0.535–0.937], FPG of < 100 mg/mL: 0.774 [95% CI 0.606–0.987]) and progression (BMI ≥ 25 kg/m^2^: 0.668 [95% CI 0.568–0.784], FPG of < 100 mg/mL: 0.732 [95% CI 0.582–0.921]). The present study did not verify a significant overall association between RASi use and NAFLD development/progression but suggested that RASi might prevent NAFLD development and progression among specific subjects.

## Introduction

Non-alcoholic fatty liver disease (NAFLD) has been increasing in recent years, accounting for 20–30% of the population in Western countries, and as such, makes it the most common type of liver disease^1^. It is a prime determinant of long-term hepatic prognosis and is associated with the development of cirrhosis and its complications, including hepatocellular carcinoma. NAFLD is expected to become the main indication for liver transplantation in the near future^2^. Although NAFLD is a growing public health concern globally, to date there is little definitive pharmacological therapy beyond lifestyle modification and so it remains a major challenge^3^. The pathological characteristics of chronic liver disease including NAFLD, incorporate oxidative stress, inflammatory markers, and enhanced fibrosis. The renin angiotensin system (RAS), which plays a crucial role in the pathogenesis of hypertension, is also associated with these characteristics^4^. Previous studies have recognized that increased activation of both hepatic and systemic RAS can contribute to the pathophysiology of liver diseases^5^. Angiotensin II actions are known to be related to the detrimental effects that influence the spectrum of histological changes observed in NAFLD^6,7^. Recently, there has been renewed interest in the therapeutic efficacy of RAS inhibitors (RASi) such as angiotensin converting enzyme inhibitors (ACE-I) and angiotensin II receptor blockers (ARB), in patients with NAFLD. However, most studies in the RAS field have focused only on progression of liver fibrosis, not NAFLD itself, and were restricted to limited study populations or rodent models^8^. Much less is currently known about the effect of RASi for specifically preventing NAFLD. Consequently, a need exists to further investigate the association between RASi use and NAFLD in a large-scale, longitudinal cohort setting. Therefore, we set out in this study to investigate the role of RASi on NAFLD development and progression in a large cohort and further attempted to identify more effective treatment options based on different types of RASi.

## Results

The baseline characteristics of cases and controls after PSM in both study cohorts are presented in Table 1. Men outnumbered women by 2744 (58.4%) to 1958 (41.6%) in the NAFLD development cohort, and 3324 (65.9%) to 1720 (34.1%) in the NAFLD progression cohort. Almost half the subjects were between 40 and 59 years old in both NAFLD development (45.6%) and progression (50.7%) cohorts. All matching variables including sex, age, income, smoking habits, alcohol consumption, physical activity, BMI, presence of abdominal obesity and comorbidities, SBP, DBP, FPG, cholesterol levels, and prescription drug use were evenly distributed between cases and controls in the NAFLD development cohort. Conversely, in the NAFLD progression cohort, subjects who developed NAFLD progression were older, had a higher BMI, WC, FPG, and were more often prescribed α-glucosidase inhibitors, β-blockers, sulfonylureas, metformin, and thiazolidinediones. Cases exhibiting NAFLD progression, also showed significantly higher rates of HTN, DM and CCI scores above two. Thus, these variables were further adjusted to balance the comparison. The frequency of RASi ever-users in the NAFLD development case and control cohorts were 10.1% (n = 238/2351) and 10.0% (n = 234/2351), respectively (Table 2). Overall, no significant association was evident between RASi use and NAFLD development (ever-user vs. never-user: OR 1.017; 95% CI 0.842–1.230). The use of RASi had no effect on NAFLD development in terms of cumulative dose, prescription period, or mean daily dose. In a subsample of 2744 men and 1958 women analyzed separately by gender, RASi use also did not influence NAFLD development. For the NAFLD progression cohort, RASi use, again, did not affect NAFLD progression (ever-user vs. never-user: aOR 0.942; 95% CI 0.803–1.105) (Table 3). Although RASi use generally did not show a significant reduction for NAFLD progression risk in men or women regardless of the dose and duration, women who were prescribed a mean daily RASi dose of 0.5 or more, but less than one, revealed a significantly lower aOR of 0.492 (95% CI 0.259–0.935). To evaluate the influence of RASi on NAFLD development/progression across different RASi types, we stratified NAFLD development/progression cases and each corresponding control according to ACEIs-only, ARBs-only, and ARB users who had switched from ACEIs (Table 4). A clear benefit of these RASi types in the prevention of NAFLD development or progression could not be identified in this analysis. The conditional logistic regression analysis suggested that ARB-only users and ACEI-only users were associated with reduced and increased risks of NAFLD development, respectively, but the associations were not statistically significant. ARB users who switched from ACEIs showed significantly higher ORs, especially in the higher cumulative RASi dose (OR 2.067, 95% CI 1.186–3.601). In the NAFLD progression cohort, after adjusting for confounders, we found that ACEI-only users ≥ 180 cDDD were less likely to have experienced NAFLD progression (aOR 0.539, 95% CI 0.293–0.992). We conducted some stratified analyses according to cDDD to determine whether the relationship between RASi use and NAFLD development/progression varied within specific clinical situations, as presented in Table 5. An additional analysis in this study included cases of individuals who were obese with BMI ≥ 25 kg/m^2^ or who had normal FPG of < 100 mg/mL. RASi ever-use in these cases was associated with reduced risk of both NAFLD development (BMI ≥ 25 kg/m^2^: OR 0.708, 95% CI 0.535–0.937, FPG of < 100 mg/mL: OR 0.774, 95% CI 0.606–0.987) and progression (BMI ≥ 25 kg/m^2^: aOR 0.668, 95% CI 0.568–0.784, FPG of < 100 mg/mL: aOR 0.732, 95% CI 0.582–0.921). A trend toward risk reduction became apparent as the cDDD went up in both groups (observed obesity/normal glucose level) of the NAFLD development cohort. In contrast, these groups within the NAFLD development cohort showed no positive correlation between increase of cDDD and risk reduction.

**Table 1:** Baseline characteristics of cases and controls after propensity score matching.

	NAFLD development			NAFLD progression		
	Cases (n = 2351)	Controls (n = 2351)	*P* value	Cases (n = 2522)	Controls (n = 2522)	*P*-value
	N (%)	N (%)		N (%)	N (%)	
**Sex**						
Men	1376 (58.5)	1368 (58.2)	0.839	1641 (65.1)	1683 (66.7)	0.223
Women	975 (41.5)	983 (41.8)		881 (34.9)	839 (33.3)	
**Age (years)**						
20–29	444 (18.9)	404 (17.2)	0.574	109 (4.3)	160 (6.3)	0.010
30–39	540 (23.0)	547 (23.3)		491 (19.5)	531 (21.1)	
40–49	594 (25.3)	630 (26.8)		627 (24.9)	638 (25.3)	
50–59	449 (19.1)	470 (20.0)		667 (26.4)	627 (24.9)	
60–69	249 (10.6)	233 (9.9)		456 (18.1)	402 (15.9)	
≥ 70	75 (3.2)	67 (2.8)		172 (6.8)	164 (6.5)	
**Income (decile)**						
1–3	571 (24.3)	567 (24.1)	0.555	531 (21.1)	515 (20.4)	0.728
4–7	1024 (43.6)	1058 (45.0)		984 (39.0)	1010 (40.0)	
8–10	756 (32.2)	726 (30.9)		1007 (39.9)	997 (39.5)	
**Smoking (pack-year)**						
None	1420 (60.4)	1425 (60.6)	0.691	1407 (558)	1382 (54.8)	0.897
Past < 10	137 (5.8)	140 (6.0)		152 (6.0)	159 (6.3)	
Past ≥ 10	130 (5.5)	139 (5.9)		327 (13.0)	341 (13.5)	
Current < 10	319 (13.6)	334 (14.2)		212 (8.4)	224 (8.9)	
Current ≥ 10	345 (14.7)	313 (13.3)		424 (16.8)	416 (16.5)	
**Alcohol consumption**						
None	1527 (65.0)	1534 (65.2)	0.854	1744 (69.2)	1760 (69.8)	0.647
Moderate^a^	824 (35.0)	817 (34.8)		778 (30.8)	762 (30.2)	
**Physical activity**						
No	531 (22.6)	537 (22.8)	0.862	668 (26.5)	659 (26.1)	0.798
Yes^b^	1820 (77.4)	1814 (77.2)		1854 (73.5)	1863 (73.9)	
**BMI (kg/m**^**2**^**)**						
< 18.5	6 (0.3)	6 (0.3)	0.810	0 (0)	0 (0)	0.001
18.5–22.9	511 (21.7)	496 (21.1)		14 (0.6)	6 (0.2)	
23–24.9	848 (36.1)	880 (37.4)		54 (2.1)	25 (1.0)	
≥ 25	986 (41.9)	969 (41.2)		2454 (97.3)	2491 (98.8)	
**WC (cm)**						
< 90 (men), < 85 (women)	2100 (89.3)	2106 (89.6)	0.812	350 (13.9)	286 (11.3)	0.008
≥ 90 (men), ≥ 85 (women)	251 (10.7)	245 (10.4)		2172 (86.1)	2236 (88.7)	
**SBP (mm Hg)**						
< 130	1634 (69.5)	1658 (70.5)	0.482	1204 (47.7)	1181 (46.8)	0.801
130–139	519 (22.1)	486 (20.7)		792 (31.4)	802 (31.8)	
≥ 140	198 (8.4)	207 (8.8)		526 (20.9)	539 (21.4)	
**DBP (mm Hg)**						
< 80	1379 (58.7)	1378 (58.6)	0.983	926 (36.7)	912 (36.2)	0.846
80–89	780 (33.2)	784 (33.3)		1109 (44.0)	1108 (43.9)	
≥ 90	192 (8.2)	189 (8.0)		487 (19.3)	502 (19.9)	
**FPG (mg/dL)**						
< 100	1778 (75.6)	1756 (74.7)	0.744	1308 (51.9)	1394 (55.4)	0.004
100–125	520 (22.1)	542 (23.1)		855 (33.9)	841 (33.3)	
≥ 126	53 (2.3)	53 (2.3)		359 (14.2)	287 (11.4)	
**TC (mg/dL)**						
< 200	1397 (59.4)	1385 (58.9)	0.896	1013 (40.2)	1009 (40.0)	0.989
200–239	707 (30.1)	710 (30.2)		945 (37.5)	945 (37.5)	
≥ 240	247 (10.5)	256 (10.9)		564 (22.4)	568 (22.5)	
**TG (mg/dL)**						
< 150	1951 (83.0)	1929 (82.1)	0.694	711 (28.2)	741 (29.4)	0.642
150–199	268 (11.4)	281 (12.0)		630 (25.0)	616 (24.4)	
≥ 200	132 (5.6)	141 (6.0)		1181 (46.8)	1165 (46.2)	
**LDL-C (mg/dL)**						
< 100	651 (27.7)	649 (27.6)	0.968	728 (28.9)	694 (27.5)	0.876
100–129	856 (36.4)	846 (36.0)		782 (31.0)	793 (31.4)	
130–159	570 (24.2)	568 (24.2)		672 (26.6)	685 (27.2)	
160–189	195 (8.3)	209 (8.9)		258 (10.2)	263 (10.4)	
≥ 190	79 (3.4)	79 (3.4)		82 (3.3)	87 (3.4)	
**HDL-C (mg/dL)**						
< 40	295 (12.5)	300 (12.8)	0.844	601 (23.8)	584 (23.2)	0.666
40–50	1467 (62.4)	1479 (62.9)		1534 (60.8)	1565 (62.1)	
≥ 50	589 (25.1)	572 (24.3)		387 (15.3)	373 (14.8)	
**Antihypertensives**						
β-Blockers	284 (12.1)	273 (11.6)	0.652	507 (20.1)	441 (17.5)	0.019
CCBs	354 (15.1)	353 (15.0)	1.000	791 (31.4)	740 (29.3)	0.126
Diuretics	309 (13.1)	306 (13.0)	0.931	693 (27.5)	655 (26.0)	0.239
**Hypoglycemic agents**						
α-Glucosidase inhibitors	14 (0.6)	15 (0.6)	1.000	118 (4.7)	71 (2.8)	0.001
DPP4i	31 (1.3)	33 (1.4)	0.900	147 (5.8)	130 (5.2)	0.323
Insulins	17 (0.7)	15 (0.6)	0.859	81 (3.2)	62 (2.5)	0.127
Meglitinides	6 (0.3)	8 (0.3)	0.789	24 (1.0)	15 (0.6)	0.198
Metformin	66 (2.8)	76 (3.2)	0.443	407 (16.1)	307 (12.2)	0.000
Sulfonylureas	54 (2.3)	56 (2.4)	0.923	358 (14.2)	257 (10.2)	0.000
Thiazolidinediones	7 (0.3)	7 (0.3)	1.000	77 (3.1)	52 (2.1)	0.032
**CCI score**						
0	635 (27.0)	617 (26.2)	0.567	429 (17.0)	452 (17.9)	0.005
1	634 (27.0)	661 (28.1)		516 (20.5)	573 (22.7)	
2	445 (18.9)	468 (19.9)		489 (19.4)	532 (21.1)	
3	289 (12.3)	289 (12.3)		384 (15.2)	372 (14.8)	
≥ 4	348 (14.8)	316 (13.4)		704 (27.9)	593 (23.5)	
**Comorbidities**						
Diabetes	69 (2.9)	71 (3.0)	0.932	425 (16.9)	116 (4.6)	0.000
Hypertension	524 (22.3)	528 (22.5)	0.916	1127 (44.7)	1049 (41.6)	0.029

^a^< 7 drinks per week for women, < 14 drinks per week for men.

^b^Moderate intensity activity ≥ 150 min per week, or vigorous intensity activity ≥ 75 min per week.

*BMI *body mass index, *WC *waist circumference, *CCBs *calcium channel blocker, *CCI *Charlson comorbidity index, *DBP *diastolic blood pressure, *DPP4i *dipeptidyl peptidase-4 inhibitors, *HDL-C *high-density lipoprotein cholesterol, *LDL-C *low-density lipoprotein cholesterol, *NAFLD *non-alcoholic fatty liver disease, *PSM *propensity score matching, *SBP *systolic blood pressure, *SD *standard eviation.

**Table 2:** Risk of NAFLD development according to the RASi exposures.

	NAFLD development	Non-NAFLD development	Conditional logistic regression					
			Total		Men		Women	
	Total (men, women)	Total (men, women)	OR (95% CI)	*p*	OR (95% CI)	*p*	OR (95% CI)	*p*
**RASi use**								
Never	2113 (1274, 839)	2117 (1264, 853)	1.00 (reference)		1.00 (reference)		1.00 (reference)	
Ever	238 (102, 136)	234 (104, 130)	1.02 (0.84–1.23)	0.859	0.90 (0.67–1.23)	0.517	1.11 (0.83–1.49)	0.488
**RASi cumulative daily defined dose (cDDD)**								
> 0–90	65 (29, 36)	69 (32, 37)	0.92 (0.65–1.31)	0.652	0.79 (0.46–1.37)	0.404	0.88 (0.49–1.56)	0.650
90–180	20 (11, 9)	20 (7, 13)	1.00 (0.54–1.86)	0.998	0.88 (0.30–2.56)	0.816	0.81 (0.30–2.18)	0.681
180–365	18 (6, 12)	28 (12, 16)	0.66 (0.36–1.20)	0.174	0.52 (0.18–1.44)	0.206	0.86 (0.36–2.06)	0.736
365–720	41 (15, 26)	28 (12, 16)	1.47 (0.91–2.39)	0.115	1.62 (0.71–3.69)	0.252	1.69 (0.82–3.45)	0.153
≥ 720	94 (41, 53)	89 (41, 48)	1.06 (0.79–1.43)	0.698	0.94 (0.59–1.51)	0.800	1.25 (0.79–1.96)	0.343
**RASi total prescription days**								
> 0–180	81 (40, 41)	94 (40, 54)	0.85 (0.63–1.15)	0.288	0.78 (0.48–1.27)	0.322	0.77 (0.46–1.27)	0.305
180–365	23 (8, 15)	20 (9, 11)	1.21 (0.67–2.21)	0.528	1.18 (0.43–3.25)	0.753	1.22 (0.51–2.93)	0.659
365–720	34 (11, 23)	27 (14, 13)	1.26 (0.76–2.10)	0.369	0.97 (0.41–2.30)	0.940	1.69 (0.79–3.61)	0.177
720	100 (43, 57)	93 (41, 52)	1.07 (0.80–1.43)	0.628	0.96 (0.60–1.52)	0.853	1.24 (0.80–1.93)	0.338
**RASi mean prescribed daily dose (PDD)**								
> 0–0.5	2 (2, 0)	3 (1, 2)	0.70 (0.12–4.22)	0.701	1.75 (0.16–19.43)	0.650	–	–
0.5–1	61 (19, 42)	50 (22, 28)	1.20 (0.82–1.76)	0.343	0.81 (0.41–1.58)	0.535	1.27 (0.73–2.21)	0.398
1–1.5	140 (68, 72)	146 (68, 78)	0.96 (0.75–1.22)	0.723	0.90 (0.62–1.32)	0.598	1.03 (0.70–1.51)	0.885
≥ 1.5	35 (13, 22)	35 (13, 22)	1.02 (0.64–1.65)	0.920	1.00 (0.42–2.34)	0.992	1.34 (0.65–2.75)	0.424

*CI *confidence interval, *NAFLD *non-alcoholic fatty liver disease, *OR *odds ratio, *RASi *renin–angiotensin–aldosterone system inhibitors.

**Table 3:** Risk of NAFLD progression according to the RASi exposures.

	NAFLD progression	Non-NAFLD progression	Conditional logistic regression					
			Total		Men		Women	
	Total (men, women)	Total (men, women)	aOR^a^ (95% CI)	*p*	aOR^a^ (95% CI)	*p*	aOR^a^ (95% CI)	*p*
**RASi use**								
Never	2001 (1334, 667)	2008 (1386, 622)	1.00 (reference)		1.00 (reference)		1.00 (reference)	
Ever	521 (307, 214)	514 (297, 217)	0.94 (0.80–1.11)	0.465	1.02 (0.82–1.26)	0.884	0.88 (0.66–1.18)	0.395
**RASi cumulative daily defined dose (cDDD)**								
> 0–90	127 (77, 50)	141 (88, 53)	0.81 (0.61–1.07)	0.136	0.82 (0.56–1.18)	0.281	0.79 (0.48–1.31)	0.365
90–180	51 (27, 24)	42 (23, 19)	1.14 (0.72–1.79)	0.576	1.28 (0.68–2.39)	0.444	1.16 (0.54–2.51)	0.705
180–365	59 (35, 24)	55 (29, 26)	1.05 (0.69–1.58)	0.832	1.28 (0.73–2.27)	0.391	1.12 (0.57–2.18)	0.751
365–720	71 (32, 39)	71 (41, 30)	0.83 (0.57–1.21)	0.335	0.77 (0.45–1.31)	0.338	1.00 (0.53–1.87)	0.989
≥ 720	213 (136, 77)	205 (116, 89)	1.01 (0.80–1.27)	0.925	1.13 (0.84–1.53)	0.427	0.77 (0.49–1.19)	0.237
**RASi total prescription days**								
> 0–180	184 (109, 75)	180 (110, 70)	0.92 (0.72–1.17)	0.510	0.96 (0.70–1.32)	0.808	0.87 (0.57–1.34)	0.540
180–365	52 (26, 26)	63 (40, 23)	0.84 (0.56–1.27)	0.404	0.72 (0.42–1.26)	0.255	1.65 (0.84–3.27)	0.148
365–720	73 (40, 33)	73 (37, 36)	0.84 (0.58–1.22)	0.369	1.04 (0.61–1.78)	0.882	0.69 (0.37–1.31)	0.260
≥ 720	212 (132, 80)	198 (110, 88)	1.03 (0.82–1.30)	0.777	1.16 (0.86–1.58)	0.338	0.79 (0.51–1.22)	0.290
**RASi mean prescribed daily dose (PDD)**								
> 0–0.5	4 (3, 1)	4 (3, 1)	1.14 (0.26–4.99)	0.862	0.98 (0.16–6.03)	0.981	–	–
0.5–1	92 (59, 33)	93 (45, 48)	0.94 (0.67–1.30)	0.694	1.52 (0.98–2.37)	0.063	0.49 (0.26–0.93)	0.030
1–1.5	336 (190, 146)	322 (182, 140)	0.96 (0.79–1.16)	0.642	0.99 (0.76–1.28)	0.929	0.94 (0.68–1.31)	0.726
≥ 1.5	89 (55, 34)	95 (67, 28)	0.89 (0.64–1.24)	0.500	0.77 (0.51–1.18)	0.232	1.16 (0.63–2.16)	0.630

^a^Adjusted for age, body mass index, waist circumference, fasting plasma glucose, α-glucosidase inhibitor, β-blocker, metformin, sulfonylurea, thiazolidinedione, Charlson comorbidity index score, diabetes, hypertension.

*CI *confidence interval, *NAFLD *non-alcoholic fatty liver disease, *OR *odds ratio, *RASi *renin–angiotensin–aldosterone system inhibitor.

**Table 4:** Risk of NAFLD development and progression according to the types of RASi.

	NAFLD development				NAFLD progression			
	Cases N	Controls N	OR (95% CI)	*p*	Cases N	Controls N	aOR^a^ (95% CI)	*p*
RASi never user	2113	2117	1.00 (reference)		2001	2008	1.00 (reference)	
**RASi user**								
ACEi only user (cDDD)								
Ever^b^ (> 0)	46	34	1.37 (0.88–2.16)	0.167	68	70	0.84 (0.57–1.22)	0.356
≥ 30	30	25	1.23 (0.72–2.11)	0.442	47	52	0.76 (0.48–1.22)	0.253
≥ 60	25	19	1.35 (0.74–2.47)	0.324	42	44	0.85 (0.52–1.40)	0.528
≥ 120	22	19	1.19 (0.64–2.21)	0.580	30	37	0.64 (0.36–1.14)	0.129
≥ 180	18	14	1.32 (0.65–2.68)	0.436	25	35	0.54 (0.29–0.99)	0.047
ARB only user (cDDD)								
Ever^b^ (> 0)	143	170	0.83 (0.66–1.05)	0.115	348	361	0.93 (0.77–1.11)	0.421
≥ 30	120	143	0.83 (0.65–1.07)	0.153	299	309	0.96 (0.78–1.17)	0.659
≥ 60	110	126	0.86 (0.66–1.12)	0.249	285	277	1.03 (0.84–1.27)	0.746
≥ 120	96	116	0.82 (0.62–1.08)	0.152	253	241	1.04 (0.83–1.29)	0.746
≥ 180	91	106	0.85 (0.64–1.13)	0.262	231	222	1.04 (0.83–1.31)	0.714
ACEi → ARB switch user^c^ (cDDD)								
Ever^b^ (> 0)	38	19	2.07 (1.19–3.60)	0.010	67	49	1.23 (0.81–1.87)	0.334
≥ 30	38	19	2.06 (1.18–3.60)	0.011	66	48	1.19 (0.77–1.84)	0.438
≥ 60	38	19	2.06 (1.18–3.60)	0.011	64	47	1.19 (0.76–1.85)	0.444
≥ 120	37	17	2.22 (1.24–3.97)	0.007	59	47	1.15 (0.74–1.80)	0.530
≥ 180	34	16	2.18 (1.20–3.99)	0.011	56	47	1.11 (0.70–1.74)	0.660

^a^Adjusted for age, body mass index, waist circumference, fasting plasma glucose, α-glucosidase inhibitors, β-blocker, metformin, sulfonylurea, thiazolidinedione, Charlson Comorbidity Index score, diabetes, hypertension.

^b^Multivariable conditional logistic regression including RASi never user, ACEi only user, ARB only user, and ACEi → ARB switch user groups.

^c^Individuals who used ACEi before the first prescription of ARB.

*ACEi *angiotensin converting enzyme inhibitor, *ARB *angiotensin receptor blocker, *cDDD *cumulative daily defined dose, *CI *confidence interval, *OR *odds ratio, *RASi *renin–angiotensin–aldosterone system inhibitors, *NAFLD *non-alcoholic fatty liver disease.

**Table 5:** Risk of NAFLD development and progression according to the RASi exposures in subgroups.

	NAFLD development				NAFLD progression			
	Cases (n = 986)	Controls (n = 986)	Conditional logistic regression		Cases (n = 2454)	Controls (n = 2454)	Conditional logistic regression	
	N	N	OR (95% CI)	*p*	N	N	aOR^a^ (95% CI)	*p*
**A. Individuals with BMI ≥ 25 kg/m**^**2**^								
RASi never user	877	847	1.00 (reference)		1953	1862	1.00 (reference)	
RASi user (cDDD)								
Ever (> 0)	109	139	0.71 (0.53–0.94)	0.016	501	592	0.67 (0.57–0.78)	< 0.001
≥ 30	97	121	0.71 (0.53–0.97)	0.028	431	510	0.67 (0.56–0.79)	< 0.001
≥ 60	87	109	0.71 (0.52–0.97)	0.032	408	464	0.70 (0.58–0.84)	< 0.001
≥ 120	79	99	0.69 (0.50–0.97)	0.031	358	415	0.69 (0.57–0.83)	< 0.001
≥ 180	72	93	0.68 (0.48–0.96)	0.029	327	391	0.69 (0.57–0.84)	< 0.001

	NAFLD development				NAFLD progression			
	Cases (n = 1778)	Controls (n = 1778)	Conditional logistic regression		Cases (n = 1308)	Controls (n = 1308)	Conditional logistic regression	
	N	N	aOR^b^ (95% CI)	*p*	N	N	OR (95% CI)	*p*
**B. Individuals with FPG < 100 mg/mL**								
RASi never user	1630	1595	1.00 (reference)		1093	1041	1.00 (reference)	
RASi user (cDDD)								
Ever (> 0)	148	183	0.77 (0.61–0.99)	0.039	215	267	0.73 (0.58–0.92)	0.008
≥ 30	119	157	0.71 (0.55–0.93)	0.013	180	227	0.69 (0.54–0.89)	0.004
≥ 60	108	144	0.70 (0.53–0.93)	0.013	172	200	0.76 (0.59–0.98)	0.038
≥ 120	97	129	0.71 (0.53–0.95)	0.019	150	180	0.75 (0.57–0.98)	0.033
≥ 180	88	118	0.69 (0.51–0.93)	0.015	144	167	0.76 (0.58–1.00)	0.048

^a^Adjusted for diabetes, α-glucosidase inhibitor, insulin, metformin, sulfonylurea, thiazolidinedione.

^b^Adjusted for age.

*cDDD *cumulative daily defined dose, *CI *confidence interval, *OR *odds ratio, *RASi *renin–angiotensin–aldosterone system inhibitor, *NAFLD *non-alcoholic fatty liver disease.

## Discussion

The present study set out to see whether RASi has any effect on hepatic steatosis and fibrosis in a large-scale population-based cohort. NAFLD encompasses a wide pathological spectrum of liver injury and has been recognized not only as a potential cause of any liver-related disorder, but also as a surrogate for the risk of cardiovascular disease^9^. It has become an increasingly major challenge for the clinician and will be a critical health issue over the next couple of decades. Thus far, in the absence of an established pharmacological therapy, treatment of NAFLD has been mainly based on lifestyle modification. Indeed, it is noted that most efforts to change health-related behaviors have usually achieved only limited success.

Prior studies have noted that RAS may be important in the pathogenesis of NAFLD through its direct actions on the steatosis and fibrogenesis of hepatic stellate cells (HSC)^10^. RASi could mitigate this process and has been proposed as a therapeutic alternative for NAFLD and non-alcoholic steatohepatitis (NASH). Additionally, it has become a major area of interest because the medications are already widely used, safe, and reasonably inexpensive. Accumulating experimental data has indicated that RASi might attenuate steatosis, lobular inflammation, and hepatic fibrosis. These effects appear to be due to reductions in oxidative stress and HSC activation, and, furthermore, to the downregulation of pro-inflammatory and fibrotic cytokines^11^.

Contrary to expectation, our study did not find any significant role for RASi in NAFLD development or progression across the entire cohort. This also accords with a previous study, which had shown no benefit of losartan (50 mg/day) on liver histology represented by NAFLD activity score, compared to placebo^12^. However, the deeper analysis undertaken here has furthered our understanding of the relationship between RASi and NAFLD. First, the most important clinically relevant finding is that RASi use significantly reduced the risk of NAFLD development and progression in obese individuals. This result became more obvious as the cumulative dose increased. Our observation is consistent with those of Munoz et al. who suggested that hepatic angiotensinogen expression is elevated in obese Zucker rats and chronic treatment with an ARB can restore hepatic intracellular signaling^13^. The RAS affects fatty acid metabolism in the liver through activation of angiotensin II receptor type 1 (AT1) or angiotensin II receptor type 2 (AT2). Angiotensin II activation of AT2 is associated with increased free fatty acids (FFAs), resulting in FFA flux to the liver and promoting increases in TG^14^.

Second, the risk of NAFLD development and progression was significantly reduced in subjects with FPG of < 100 mg/mL. This was unanticipated and contradicts the findings that increased angiotensin II activity contributes to altered glucose metabolism leading to insulin resistance. It is difficult to explain this result and these data must therefore be interpreted with caution. A plausible explanation may be that there are interactions, besides insulin resistance itself, between genetic, environmental, and behavioral risk factors including patatin-like phospholipase domain-containing 3 (PNPLA3) polymorphisms, diet, altered metabolic profile and NAFLD development/progression. Thus, we need to consider that insulin resistance can clearly influence RAS activation in some, but not all individuals. Additionally, plasma levels of C-reactive protein (CRP), tumor necrosis factor-α (TNF-α), and interleukin-6 (IL-6) are elevated in subjects with type 2 diabetes, indicating the existence of low-grade systemic and hyperglycemia-induced inflammation^15^. In general, it has been suggested that chronic low-grade systemic inflammation plays a key role in the progression from hepatic steatosis to NASH and fibrosis^16^. These are a likely cause of NAFLD development/progression and could explain our result regardless of increased RAS activity in subjects with insulin resistance. Further, the possibility that different concomitant pharmacological therapies or comorbidities in patients with prediabetes or diabetes might potentially affect NAFLD development/progression cannot be ruled out.

The third finding is that ACEI had a greater impact on lowering the risk of NAFLD progression, compared to other types of RASi. ACEI use significantly reduced the risk of NAFLD progression in cases with cumulative exposure greater than or equal to 180 cDDD. There have been no controlled studies which compared the effectiveness of ACEI and ARB on NAFLD progression. Thus, this observation requires further attention and cannot be extrapolated generally. It seems plausible to hypothesize that ACEI might provide additional hepatoprotective effects through elevations in bradykinin as well as through the activation of B2-type bradykinin receptors. The cumulative degree of exposure to ACEI takes on particular importance when we postulate that RASi might require a certain induction dose before its antifibrotic effects influence liver fibrosis progression. One unexpected outcome is that the RASi use significantly reduced the risk of NAFLD progression in women, but only when the PDD was greater than or equal to 0.5 and less than one. Unlike for men, there was a tendency to increase risk as the PDD went up, but this association was not statistically significant. The reason for this gender variation is not clear but it may be attributable to the existence of gender-dependent differences in hepatic ACE expression with an enhanced hepatic ACE/RAS axis in men compared to women^17^.

Most prior studies that have noted the roles of RAS in liver fibrosis and the antifibrotic effects of RASi were based on experimental rodent models so generalization of those results to human is subject to certain limitations. Therefore, the findings from our human-based study make several contributions to the current literature. First, the study is derived from a large, representative, nationwide data registry, which reflects real-world clinical circumstances with a relatively low attrition rate. The extent of this registry source mitigated the recall bias for assessment of RASi exposure data before NAFLD development/progression. We were also able to retrieve detailed prescription records, which are more reliable than self-reported data, particularly when identifying the subjects’ RASi treatment types throughout the study period. Second, we chose a nested case–control study and adopted a conditional logistic regression model to reduce potential confounding effects. Therefore, the current study design was expected to provide sufficient statistical power even with the additional analysis of certain populations with specific characteristics.

Our study has several limitations. First, the NHIS-NSC data did not include verification of compliance with RASi use. Moreover, it did not record information about noninvasive assessment of liver steatosis or fibrosis such as ultrasound, controlled attenuation parameter (CAP), transient elastography and magnetic resonance elastography. We used laboratory panel markers as our predictive tool in assessing liver steatosis and fibrosis. Thus, these findings may not achieve the same level of accuracy as histologic or imaging diagnostic modalities and the staging of the degree of fibrosis was not possible. Another important problem is that follow-up period to assess NAFLD progression was only 5–6 years in our cohort. A note of caution is due here since advanced fibrosis usually develops over a long time, beyond the timeframe of the study. Second, we realized there is evidence supporting different ethnic susceptibility for NAFLD and that the average BMIs of Korean and non-Hispanic whites in Europe are different^18^. Therefore, cautious extrapolation beyond the Korean populations should be exercised. Hepatic steatosis index (HSI), developed using a Korean cohort, may seem more obvious as an indicator in a Korean-population based study than FLI^19^. Therefore, that validation of the study results using this different fatty liver prediction model, HSI, should be addressed in future studies. Third, the present study assessed significant liver fibrosis using a single fibrosis prediction score, the BARD. We recognized that the generalizability of these results is limited. Validating our results using other fibrosis scoring systems such as NAFLD fibrosis score (NFS) or Fibrosis-4 (FIB-4) would indeed strengthen the implications of our results. However, the absence of data such as albumin and platelet counts, which were not included in this health screening program data provided by the NHIS, made that option unfeasible.

The BARD score consistently reported a high negative predictive value, ranging 81–97%, but its positive predictive value is lower than that of other clinical models^20^. Noninvasive composite scoring systems are not liver-specific, are influenced by comorbid conditions, and limited by their low-sensitivity^21,22^.

Although liver biopsy is considered as the gold standard for determining the severity of fibrosis, it is sometimes limited by its invasiveness in clinical practice. This has increasingly led to noninvasive means of assessing fibrosis, such as the BARD score, NFS, and FIB-4. NFS and FIB-4 are accurate diagnostic modalities in exclusion of advanced fibrosis. It has also been shown that they can be safely used in patients with normal and elevated transaminase levels without showing a significant difference in diagnostic performance^23^. In patients with diabetes mellitus, they also performed adequately in terms of excluding advanced fibrosis^24^. However, in some specific populations their use remains limited. For example, in lean and obese patients, their use did not appear clinically useful^25^. In patients younger than 35 years, it has been suggested that those scores performed poorly for a diagnosis of advanced fibrosis. Moreover, different cut-offs were recommended for patients over 65 years^26^. The varying adequacy of these composite scoring systems suggests that their main clinical utility, in patients with NAFLD, lies in their ability to exclude, rather than identify, advanced fibrosis^27^. Fourth, the current study was a NHIS-NSC data sets-based, cohort study, thus, we could not evaluate the specific indications for initiating RASi treatment. However, because the Korean NHIS applies strict guidelines for reimbursement of RASi use, it is reasonable to assume that most subjects on RASi were treated according to the American College of Cardiology/American Heart Association guidelines. Fifth, we were unable to address the effect of different forms of RASi on NAFLD development/progression because of the nature of the data set. Further, the study did not evaluate the other interventions potentially related to natural history of NAFLD, such as the use of vitamin E or statin in addition to RASi treatment.

Whilst this study did not verify general benefits of RASi use on NAFLD development or progression, it did substantiate the role of RASi in specific clinical situations and offers valuable insight into the clinical implications of RASi on NAFLD.

## Conclusion

The present study could not confirm that overall, RASi use is associated with a decreased risk of NAFLD development or progression. However, these nationwide population-based real-world data suggest that RASi might prevent NAFLD development and progression among subjects with obesity or those with normal glucose level. In particular, in obese subjects, the beneficial effect of RASi showed dose-dependent tendencies. The issue of the long-term effect of RASi on NAFLD is an intriguing one which should be explored and assessed in further research.

## Methods

### Data source

The present study is based on the National Health Insurance Service-National Sample Cohort (NHIS-NSC) database version 2.0, which was updated in 2017. This large-scale cohort has been described in detail elsewhere^28–30^. In summary, NHIS-NSC is a representative nationwide (2%), stratified, random sample of the total National Health Insurance members of South Korea in 2006 with 1,108,369 subjects. This database contains 14 years of information about health care utilization (2002–2015), including age, gender, participant’s eligibility status, region, and income level, diagnostic codes, procedures, and drug prescriptions. Diagnoses were coded based on the International Statistical Classification of Disease and Related Health Problems, 10th revision (ICD-10). The NHIS provides universal health care to approximately 97% of the Korean population. Health screening is one of its services, and involves a biennial medical check-up, comprised of a self-reported questionnaire on health behavior and medical history, measurements such as height, weight, and blood pressure, and blood tests for insured people aged 40 years or older. The participation rate of the eligible population in the NHIS health screening program was 74.8% in 2014^31^. To ensure continuity of variables, specifically triglycerides, we used data for participants who underwent a national health checkup beginning January 2009 to December 2010, and who had at least one checkup after that between January 2011 and December 2015.

The NHIS approved the use of selective and anonymized data for the present study (NHIS-2019-2-198). This study protocol was approved by the Ethics Committee of Pusan National University and conducted in accordance with the principles of the Declaration of Helsinki. Because the study is based on the retrospective analysis of existing anonymous administrative and clinical data, the requirement of obtaining informed subject consent was waived by the Institutional Review Board of Pusan National University (PNU IRB/2019_63_HR).

### Study design and population

**Figure 1:**
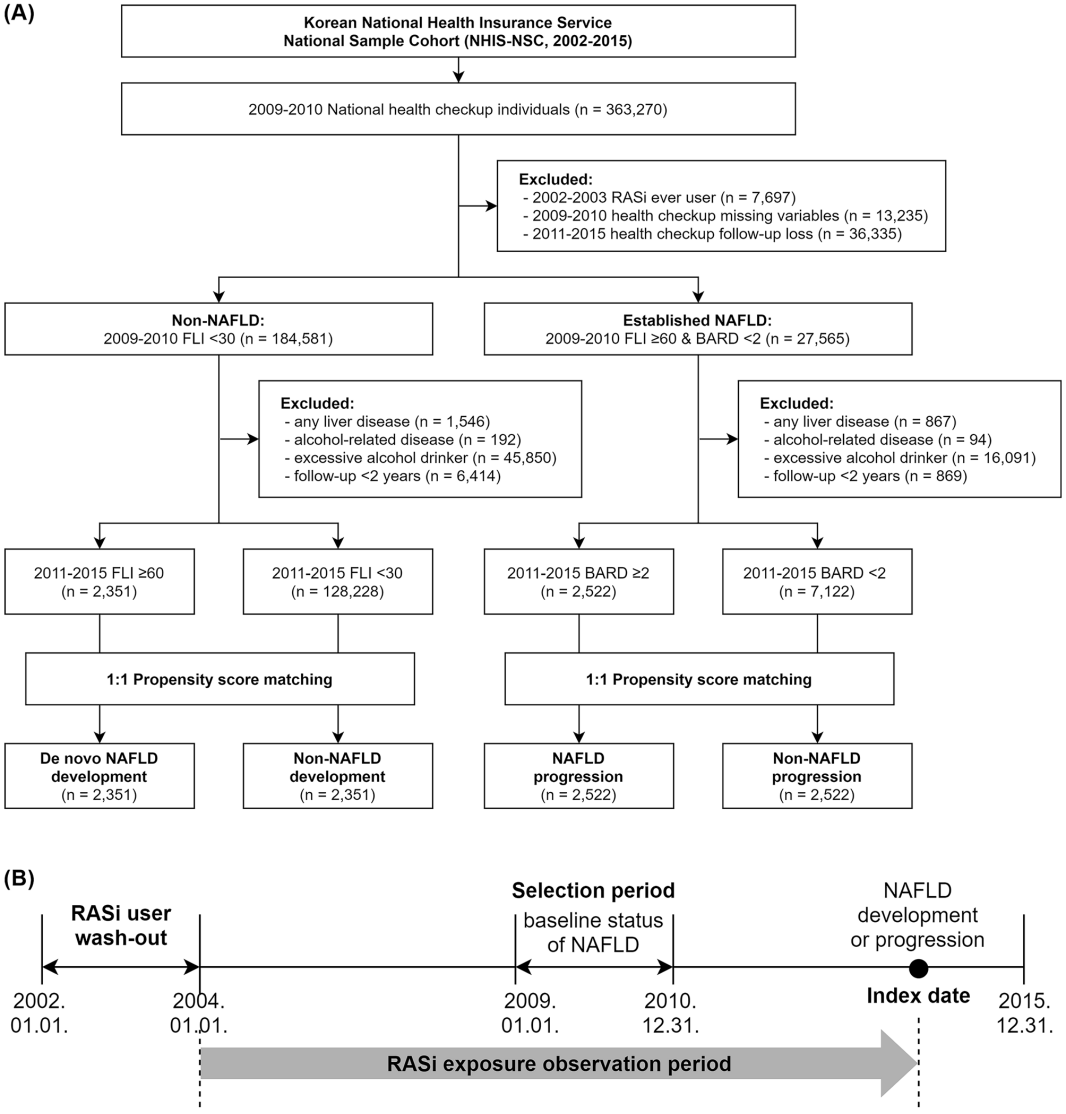
Diagrams depicting the nested case–control study design. (**A**) Flow chart of inclusion and exclusion criteria of the study population. (**B**) Timeline of the study.

For the nested case–control study design, we selected 363,270 individuals from the NHIS–NSC database who underwent the national health checkup from January 2009 to December 2010. The date of the national health checkup during that period, was considered the cohort baseline selection date (Fig. 1). We excluded subjects who met the following criteria: a record of RASi prescription (RASi ever used) in 2002 or 2003 (n = 7697); or missing data from a health checkup on any of the study related covariates (n = 13,235) and no further health checkups from 2011 to 2015 (n = 36,335). Then, subjects were classified into two groups according to NAFLD status determined by fatty liver index (FLI). The FLI is considered a surrogate marker of NAFLD based on patient BMI, levels of triglyceride and γ-glutamyl transferase (GGT), and waist circumference for referral to ultrasonography. It is calculated as: FLI = (e 0.953*loge (triglycerides) + 0.139*BMI + 0.718*loge (GGT) + 0.053*waist circumference − 15.745)/(1 + e 0.953*loge (triglycerides) + 0.139*BMI + 0.718*loge (GGT) + 0.053*waist circumference − 15.745) * 100. For FLI scores below 30, between 30 and 60, and above 60, the presence of fatty liver is considered ruled out, inconclusive, or present, respectively^32^. Thus, participants with an FLI of less than 30 at the baseline selection date, were assigned to the non-NAFLD group (n = 184,581). Subsequently, subjects were excluded if they had underlying liver disease other than NAFLD (code K76.0) before the index date (n = 1546); had alcohol-related disease before the index date (n = 192); exhibited excessive alcohol consumption (alcohol intake ≥ 30 g/day for men and ≥ 20 g/day for women, n = 45,850) during the entire study period; or if there was insufficient follow-up duration (< 2 year, n = 6414). All patients in the non-NAFLD group were divided into two study categories: those that developed NAFLD (n = 2351) and those that didn’t (n = 128,228). We defined NAFLD development (de novo NAFLD) to have occurred when participants who belonged to the non-NAFLD group at cohort entry date exhibited an FLI value of 60 or greater in any subsequent health checkup between January 2011 and December 2015. Because we excluded study participants with excessive alcohol consumption or other chronic liver disease, subsequent cases where fatty liver developed at follow-up were considered as newly developed NAFLD. Conversely, non-NAFLD development was defined as the maintenance of an FLI value below 30 for that same period. We identified 2351 subjects, who developed NAFLD, as our case group. The cohort index date of the case group was defined as the health checkup date when NAFLD development was first found. The index date of NAFLD cases were randomly assigned to the non-NAFLD controls. Of 128,228 subjects who met all criteria for non-NAFLD cases, we selected the control group using 1:1 propensity score matching (PSM). The following potential confounders were included in the matching process: age, sex, income, smoking status, alcohol consumption, physical activity, body mass index (BMI), waist circumference, systolic blood pressure (SBP), diastolic blood pressure (DBP), fasting plasma glucose (FPG), total cholesterol (TC), triglycerides (TG), low density lipoprotein cholesterol (LDL-C), high density lipoprotein cholesterol (HDL-C), hypertension, diabetes, use of antihypertensives, use of hypoglycemic agents, and Charlson Comorbidity Index (CCI) score. Ultimately, a total of 2351 de novo NAFLD development cases and 2351 controls were analyzed to determine associations between RASi use and NAFLD development.

For the analysis of NAFLD progression, participants with established NAFLD i.e. those with an FLI of 60 or greater at the baseline selection date, were selected. Among these subjects, BARD score, a well validated and surrogate marker for liver fibrosis, was used to rule out patients with advanced fibrosis. BARD score is composed of three variables and ranges from zero to four points: AST/ALT ratio ≥ 0.8 is worth two points; a BMI ≥ 28 kg/m^2^, one point; and the presence of diabetes mellitus (DM), also one point^33^. The subjects were assigned to the established NAFLD group, when they had FLI scores of 60 or greater and a BARD score less than two at the baseline selection date (n = 27,565). After applying the same exclusion criteria as for the analysis of NAFLD development, the remaining subjects in the established NAFLD group were divided into two study categories: NAFLD progression (n = 2057) and non-NAFLD progression (n = 18,607). We defined advanced liver fibrosis using a BARD score ≥ 2. Therefore, the present study assessed NAFLD progression, as exemplified by advanced fibrosis in subjects with established NAFLD, to be when participants with BARD scores < 2 at the baseline selection date exhibited a BARD score ≥ 2 in any subsequent health checkup during the study period.

Conversely, non-NAFLD progression was defined as a BARD score that stayed below two for the same period. Further, a total of 3658 NAFLD progression cases and 3658 matched controls were analyzed to determine associations between RASi use and NAFLD progression through PSM using the same process of evaluation for NAFLD development.

### Study outcomes and assessment of RASi use

The primary outcome of this study was NAFLD development or advanced fibrosis represented by NAFLD progression, among drug-naïve patients who started taking a RASi. To ascertain the roles of RASi on both NAFLD development and progression, we examined RASi exposures on the basis of prescribed medications during the observation period from January 1, 2004 to the index date. A washout period of two years prior to 2004 (2002–2003) was set to eliminate any carryover effect of RASi. RASi use was confirmed based on the prescription records in the NHIS-NSC, and we included any RASi pharmaceutical products with a history of marketing in South Korea according to the World Health Organization Collaborating Centre (WHOCC)-Anatomical Therapeutic Chemical (ATC) codes (https://www.whocc.no/atc_ddd_index/). Each individual was classified depending on their RASi exposures as ever-used (at least one prescription during the observation period) or never-used (no prescription during the observation period). The risk of NAFLD development or progression was expressed as cases per population in RASi ever-users versus never-users.

The secondary outcome measures included an odds ratio (OR) for NAFLD development or progression according to cumulative exposed amount, cumulative duration, and average prescribed dose of RASi. These were calculated to identify the associations between the intensity of RASi dosage in both NAFLD development and progression. To establish the RASi effects related to dose, we assessed an overall amount of RASi exposure using the cumulative defined daily dose (cDDD), which was calculated as the sum of the DDD for all prescribed days. This method was designed to determine the association between intensity of cumulative RASi dose and NAFLD development/progression. In addition, cumulative prescription days were obtained to identify the exposure period for RASi. We also calculated the mean prescribed daily dose (PDD), which is the average daily RASi dose dispensed to the subject regardless of RASi exposure period, to investigate the usual daily amount of RASi exposure. The definitions of DDD and PDD follow criteria published by the WHO^34^.

### Measurement and parameter definition

The characteristics of cases and controls were identified based on the results of health checkup at the selection date. The presence of comorbidities were identified based on medical records, and the use of medications was confirmed when more than 30 days of prescriptions were identified, prior to the index date. In the case of insulin, three or more pre-filled syringes were considered as equal to, or greater than a 30-day supply. NHIS-NSC database included a self-administered questionnaire covering smoking, alcohol consumption, physical activity and medical history of DM, dyslipidemia, hypertension, and malignant disease. Tobacco smoking was categorized as one of five groups according to the current smoking status and cumulative pack-years. Alcohol consumption was categorized as none or moderate (< 20 g/day in men, < 10 g/day in women). Regular exercise was defined as 150 min or more of moderate intensity activity per week, or 75 min or more of vigorous intensity activity per week. It also included anthropometric parameters such as weight, body height, and waist circumference, systolic blood pressure (SBP), and diastolic blood pressure (DBP). Body mass index (BMI) was calculated as weight/height^2^ (kg/m^2^). The participants were categorized into four BMI groups as underweight (BMI 18.5), normal (18.5–22.9), overweight (23–24.9) or obese (≥ 25.0) according to the Asian-Pacific cutoff^35^. Abdominal obesity was defined as a waist circumference (WC) ≥ 90 cm in men and ≥ 85 cm in women in accordance with the definition of the Korean Society for the Study of Obesity (KSSO)^36^.

Serum levels of FPG, TC, TG, LDL-C, and HDL-C, serum creatinine, aspartate aminotransferase (AST), alanine aminotransferase (ALT), and gamma glutamyl transpeptidase (r-GTP) were measured. The levels of all were categorized into three or four groups, respectively [FPG (mg/dL): normal < 100, pre-diabetes 100–125, diabetes ≥ 126; TC (mg/dL): desirable < 200, borderline-high 200–239, high ≥ 240; TG (mg/dL): desirable < 150, borderline-high 150–199, high ≥ 200; LDL-C (mg/dL): optimal < 100, near optimal 100–129, borderline-high 130–159, high 160–189, very high ≥ 190; HDL-C (mg/dL): low < 40, medium 40–50, high, optimal ≥ 50]. Charlson Comorbidity Index (CCI), which included a total of 17 comorbidities was evaluated based on the International Statistical Classification of Disease and Related Health Problems, 10th Revision (ICD-10).

### Statistical analysis

To adjust imbalances in the weighting of multiple confounders amongst subjects in the two study cohorts, NAFLD development/progression cases and controls were matched respectively by the propensity scores. The standardized difference was used to quantify differences in means or prevalence rates between case–control groups for continuous or categorical variables. Matching was performed using nearest neighbor matching without replacement, with each individual that developed or progressed with NAFLD being matched to one individual without^37^. Baseline data classified by two study cohorts into case–control groups were summarized as frequency (percent) after PSM. Chi-square test was used for categorical variables between the case and control groups. A conditional logistic regression analysis was conducted to estimate the associations between RASi exposure and NAFLD development/progression, and ORs and 95% confidence intervals (CIs) were measured. The NAFLD development cohort was well matched for all confounders and no further adjustment was performed. However, in the analysis of the cohort that exhibited NAFLD progression, additional adjustments for potential confounders showing significant differences between cases and controls after PSM, were conducted. Hence, age, BMI, waist circumference, FPG, use of α-glucosidase inhibitors, β-blockers, metformin, sulfonylureas, and thiazolidinediones, CCI score, and presence of DM and hypertension were adjusted in the regression analysis, and adjusted odds ratios (aORs) and 95% CIs were calculated. In the subgroup analysis, baseline characteristics of the cases and controls were compared and variables exhibiting significant differences were adjusted. A two-sided P-value < 0.05 was considered statistically significant. Statistical analyses were carried out using SAS, version 9.4 (SAS Institute, Cary, North Carolina, USA) and R version 3.1.0 (Vienna, Austria; http://www.R-project.org/).
